# Supplementary figures and images for: Deep sleep homeostatic response to naturalistic sleep loss
Source: PLOS Digit Health. 2025 Oct 6;4(10):e0001021. doi: 10.1371/journal.pdig.0001021 (PMC12500159; doi:10.1371/journal.pdig.0001021)

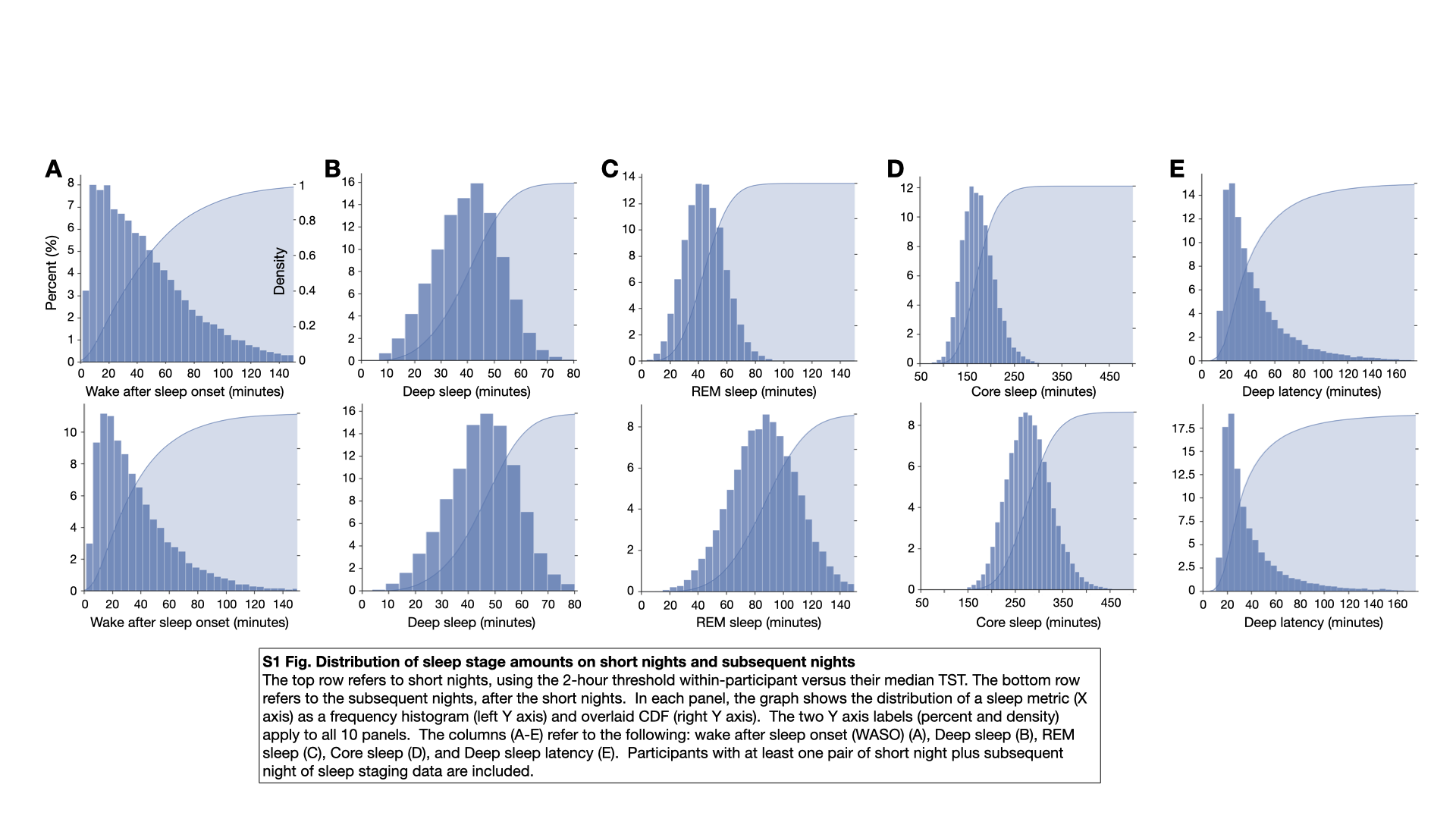

Supplement: S1 Fig — (TIFF) [file pdig.0001021.s001.tiff]

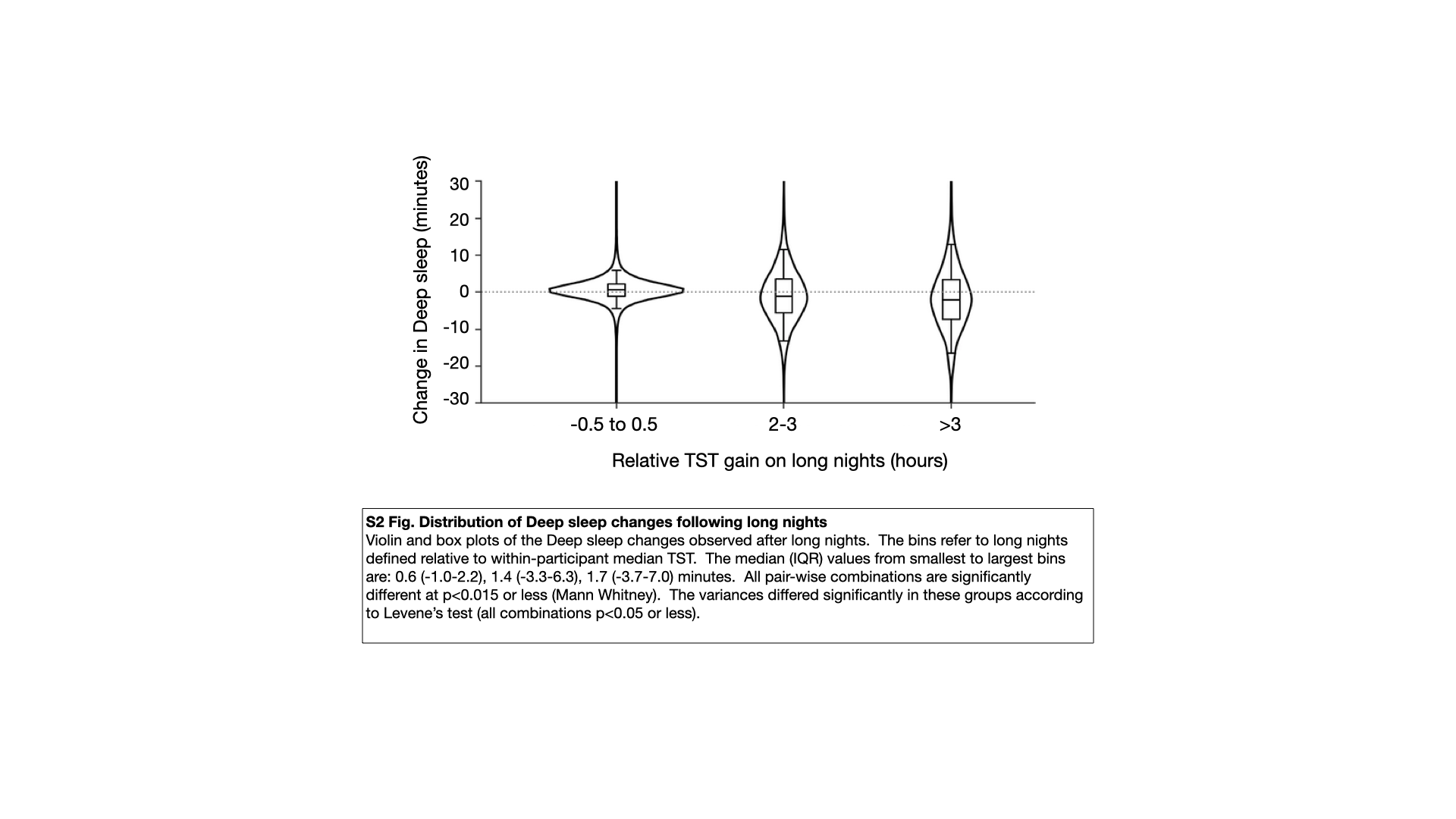

Supplement: S2 Fig — (TIFF) [file pdig.0001021.s002.tiff]

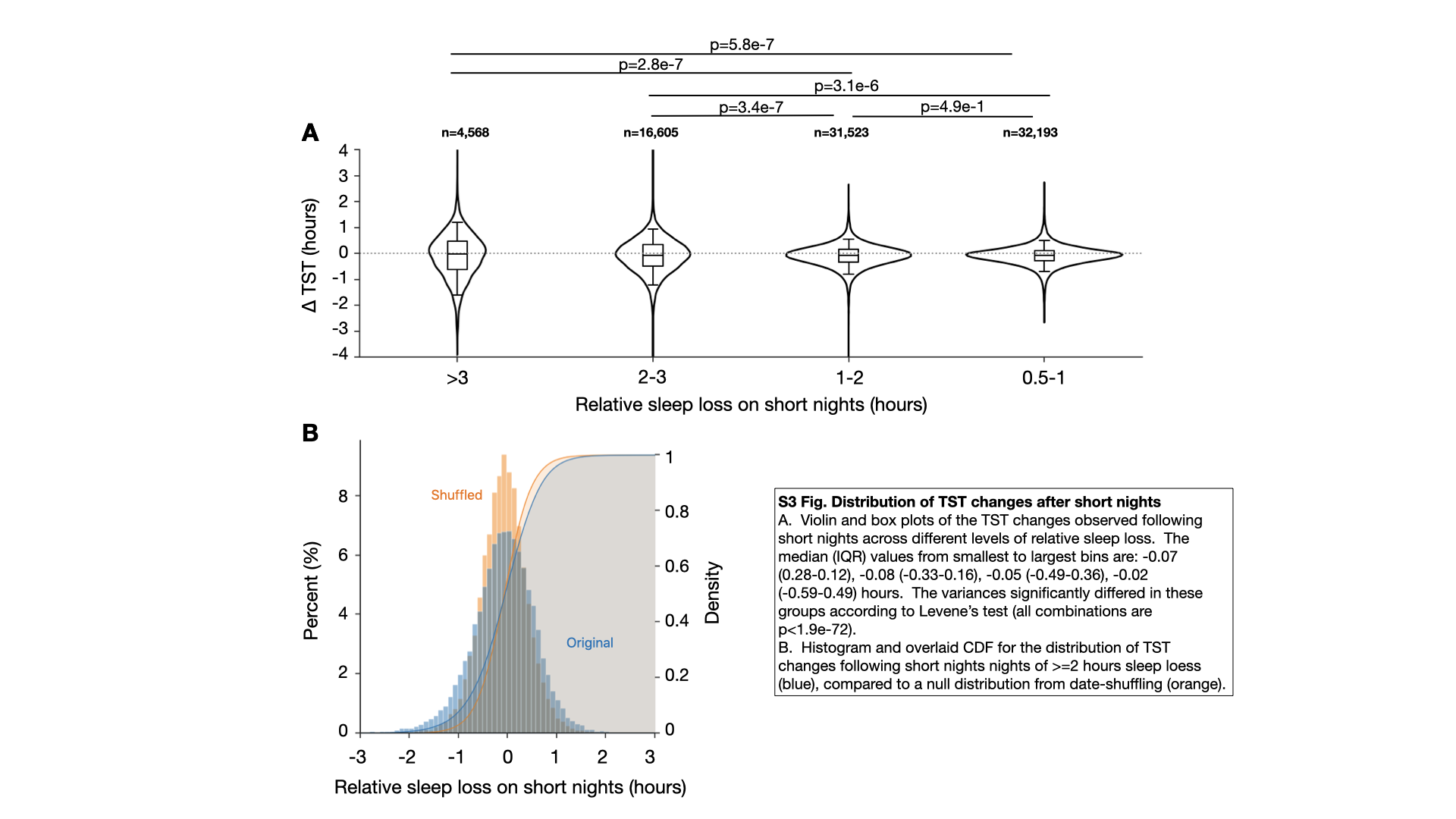

Supplement: S3 Fig — (TIFF) [file pdig.0001021.s003.tiff]

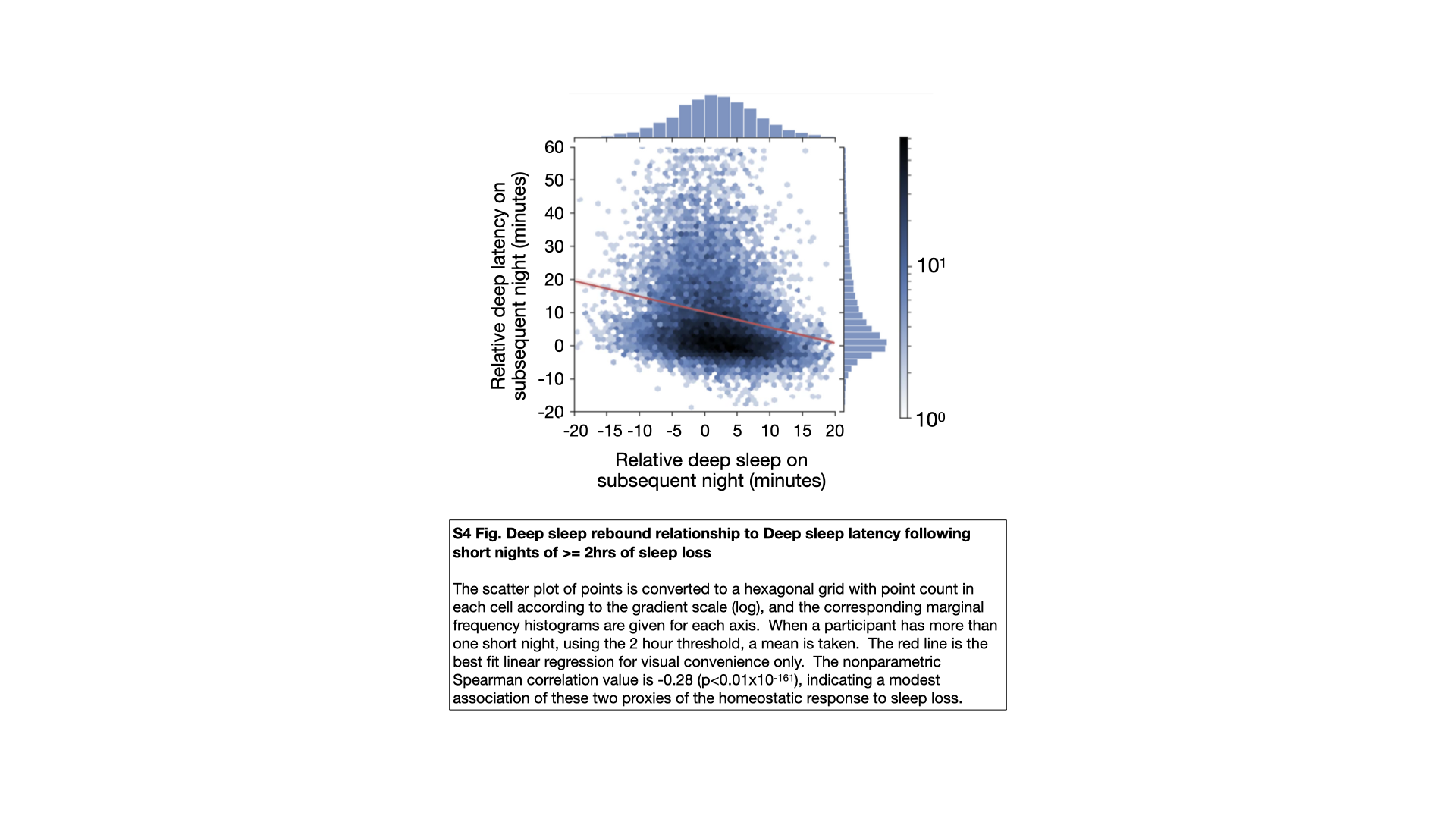

Supplement: S4 Fig — (TIFF) [file pdig.0001021.s004.tiff]

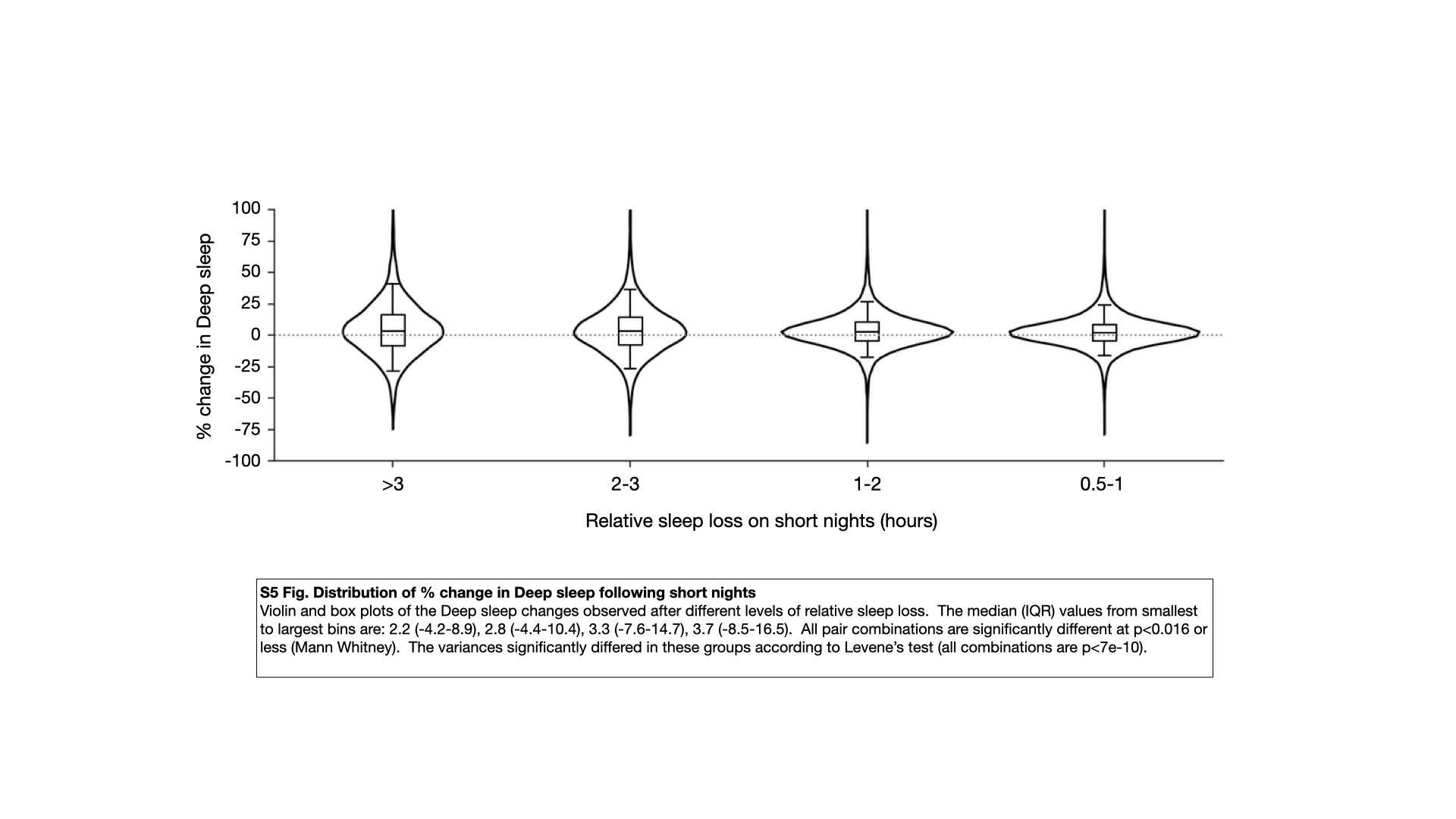

Supplement: S5 Fig — (TIFF) [file pdig.0001021.s005.tiff]

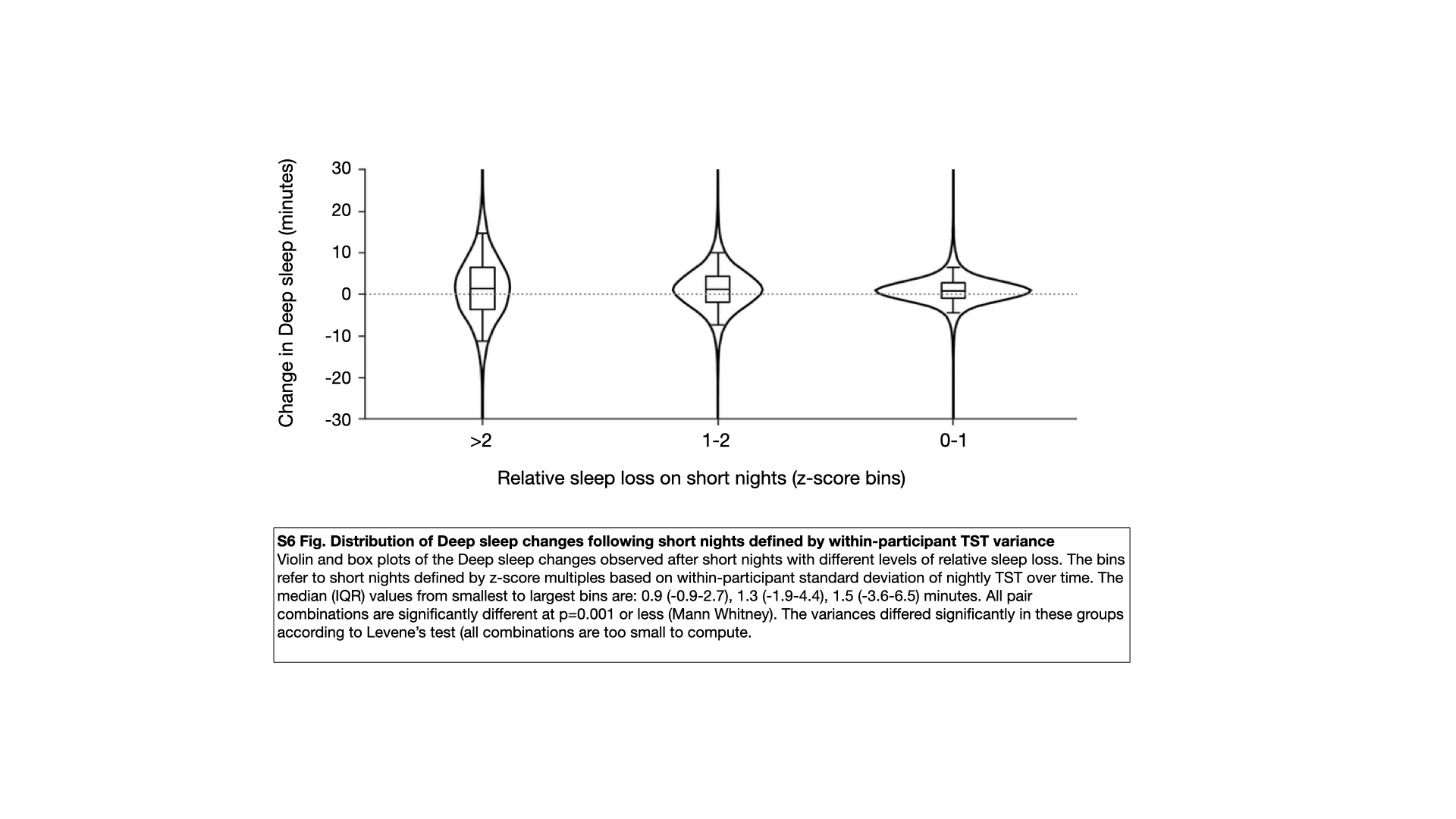

Supplement: S6 Fig — (TIFF) [file pdig.0001021.s006.tiff]

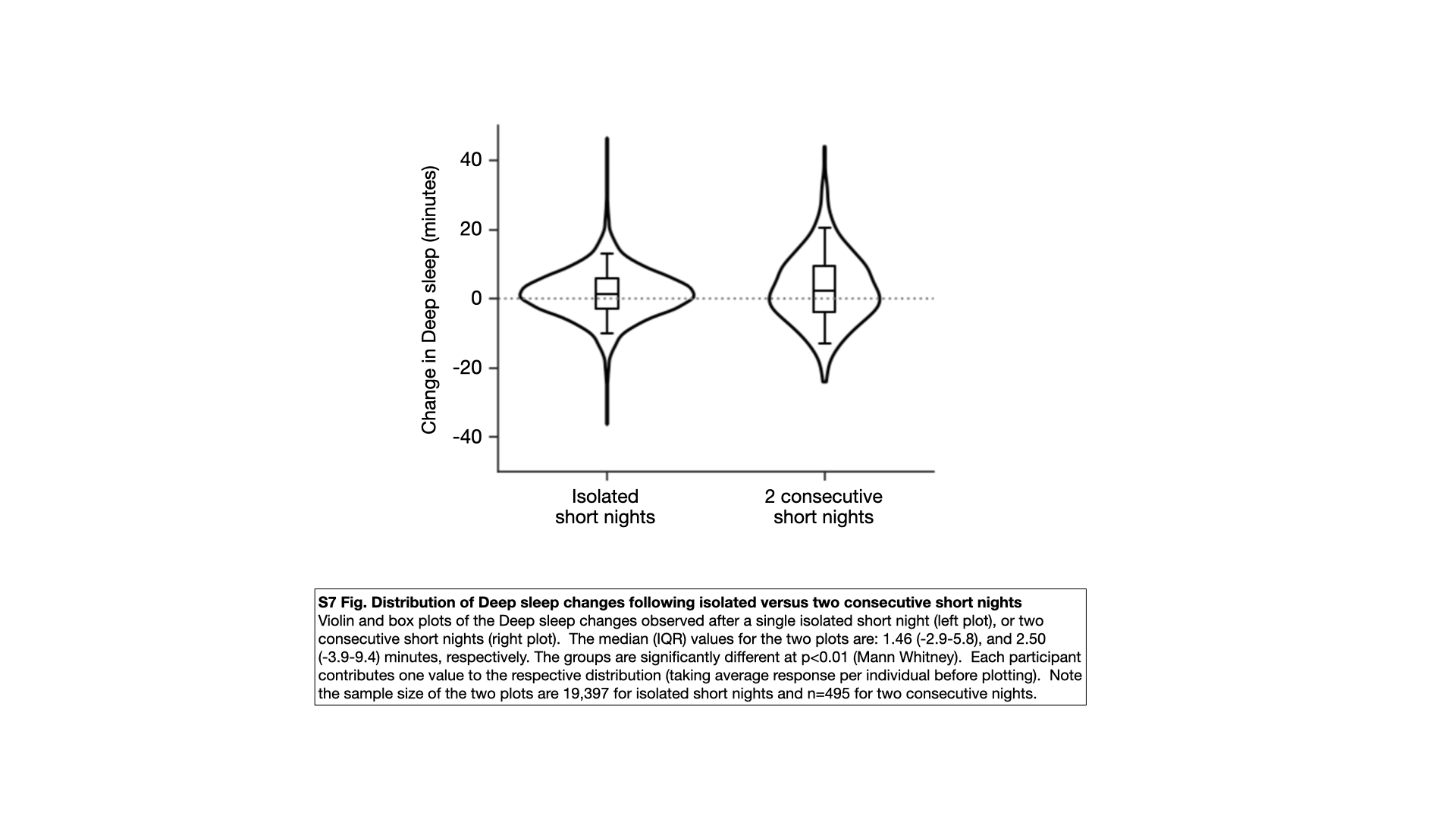

Supplement: S7 Fig — (TIFF) [file pdig.0001021.s007.tiff]

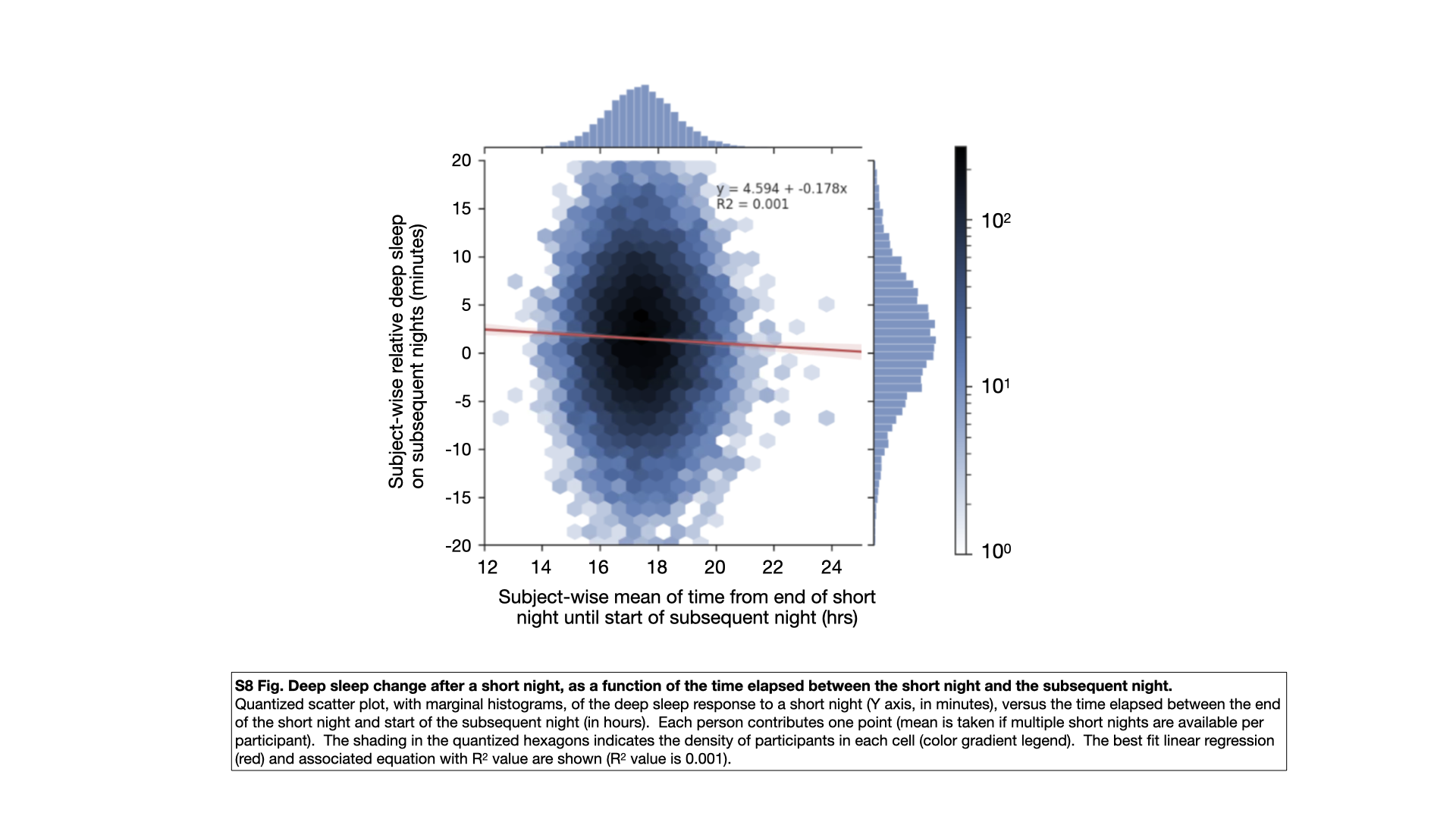

Supplement: S8 Fig — (TIFF) [file pdig.0001021.s008.tiff]

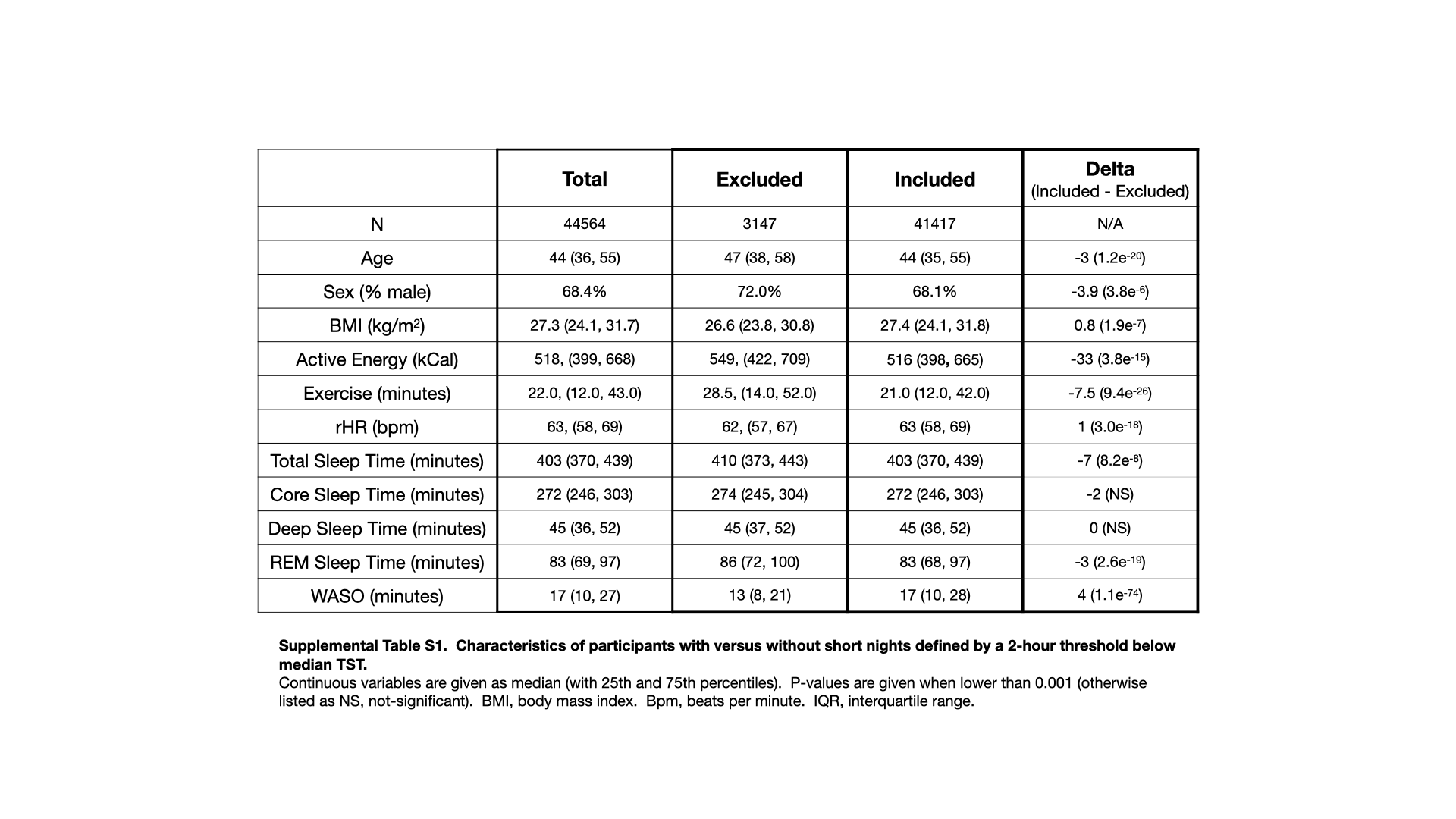

Supplement: S1 Table — (TIFF) [file pdig.0001021.s009.tiff]

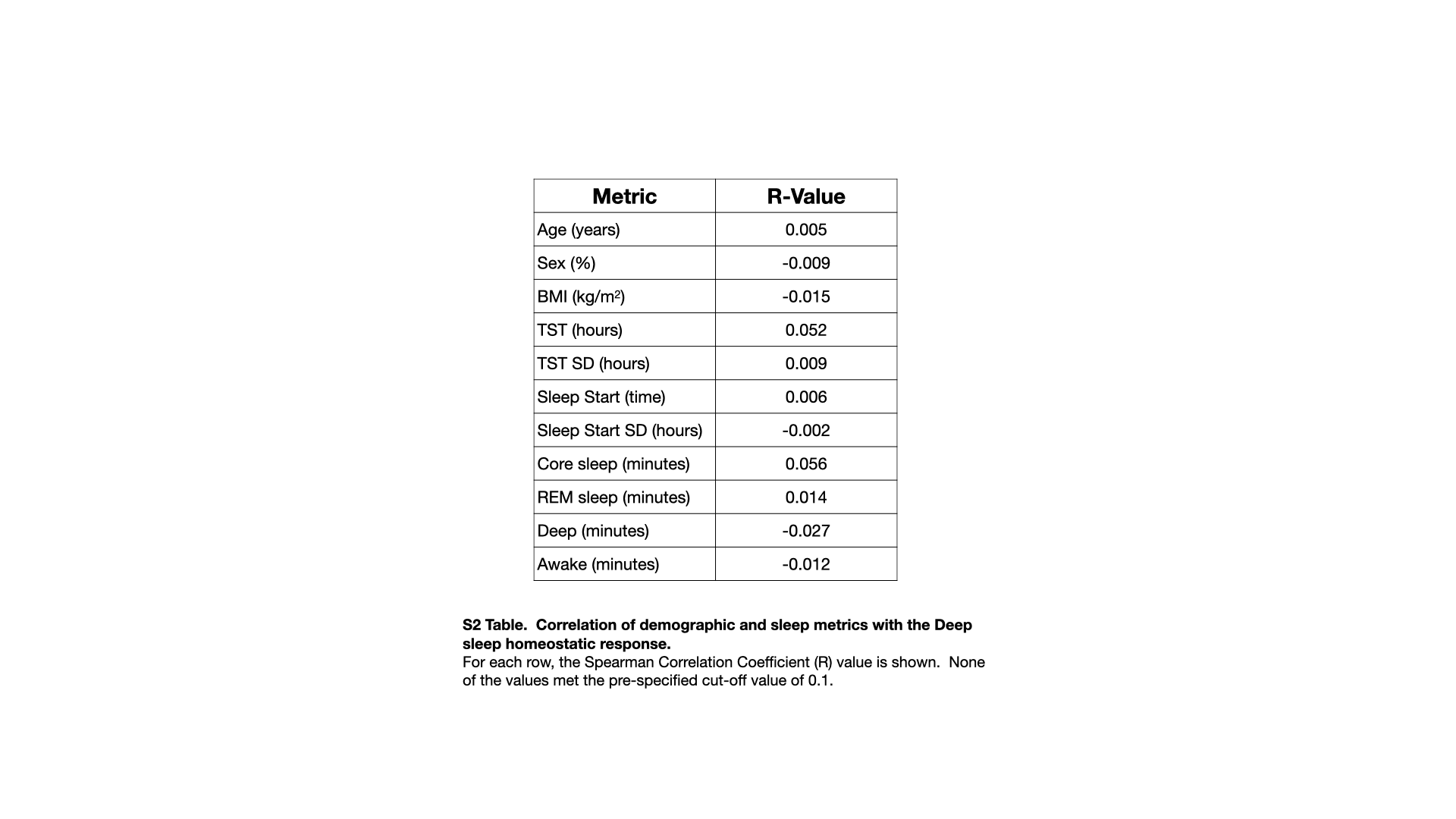

Supplement: S2 Table — (TIFF) [file pdig.0001021.s010.tiff]

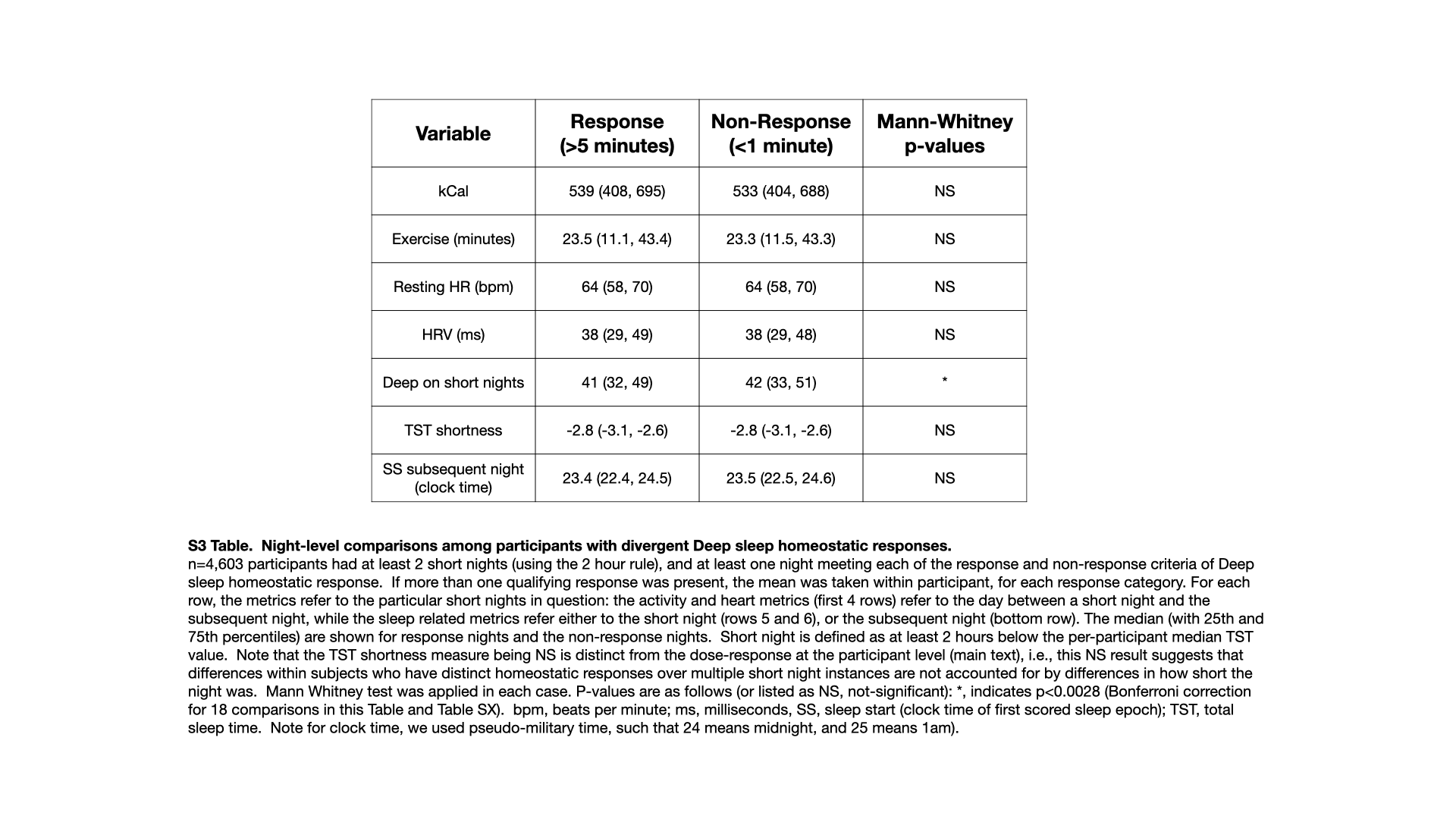

Supplement: S3 Table — (TIFF) [file pdig.0001021.s011.tiff]

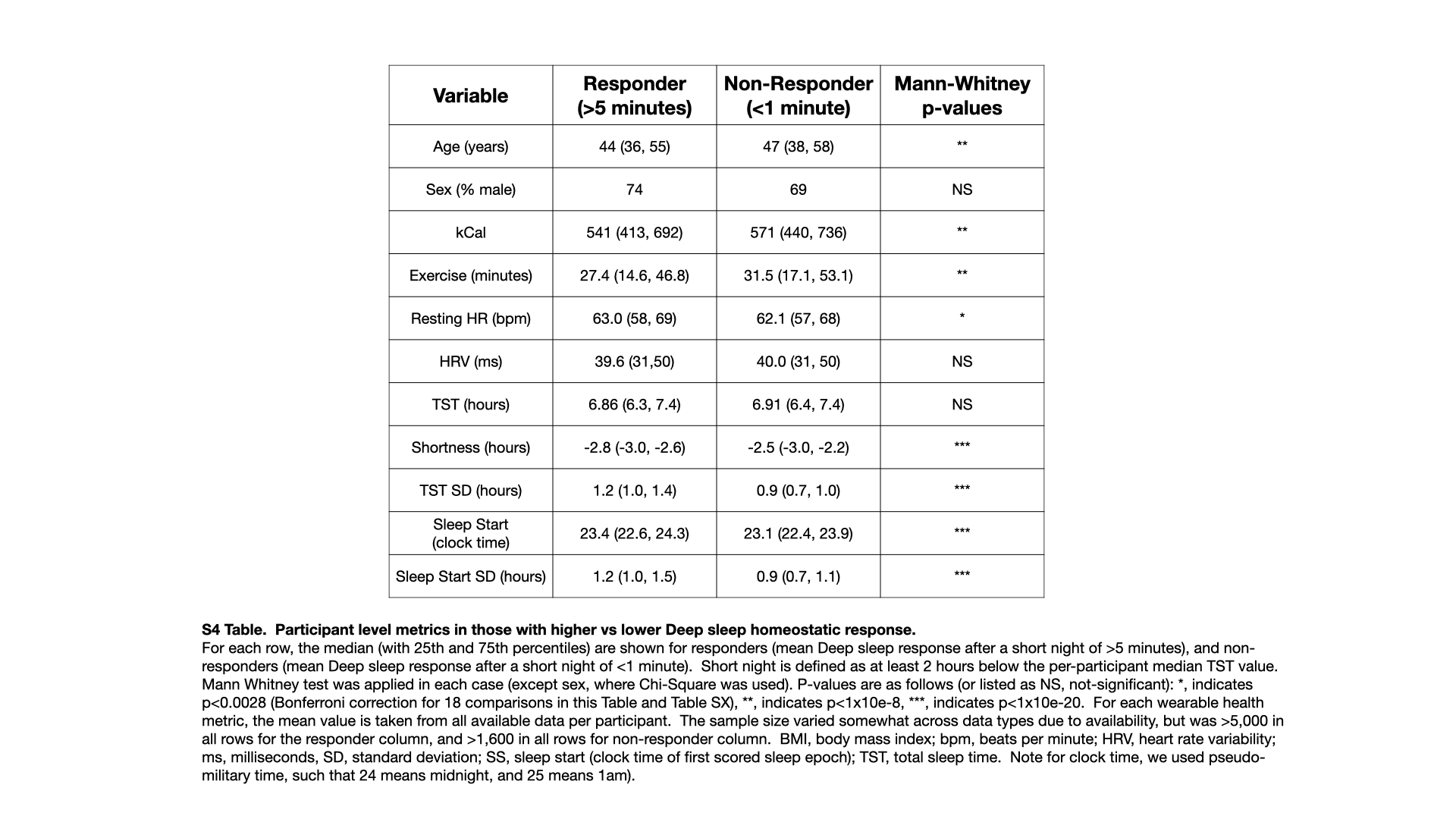

Supplement: S4 Table — (TIFF) [file pdig.0001021.s012.tiff]
